# A Comparative Study of Traditional Sun Drying and Hybrid Solar Drying on Quality, Safety, and Bioactive Compounds in “Pingo de Mel” Fig

**DOI:** 10.3390/antiox14030362

**Published:** 2025-03-19

**Authors:** Bárbara R. Henriques, Cláudia M. B. Neves, Marwa Moumni, Gianfranco Romanazzi, Carine Le Bourvellec, Susana M. Cardoso, Dulcineia F. Wessel

**Affiliations:** 1 CERNAS, Polytechnic University of Viseu, 3504-510 Viseu, Portugal; barbararhenriques@ua.pt (B.R.H.); cmneves@esav.ipv.pt (C.M.B.N.); 2 LAQV-REQUIMTE, Department of Chemistry, University of Aveiro, 3810-193 Aveiro, Portugal; 3 Department of Agricultural, Food and Environmental Sciences, Marche Polytechnic University, 60131 Ancona, Italy; m.moumni@staff.univpm.it (M.M.); g.romanazzi@univpm.it (G.R.); 4 INRAE, Avignon Université, UMR SQPOV, F-84000 Avignon, France; carine.le-bourvellec@inrae.fr

**Keywords:** *Ficus carica*, drying, food safety, phenolic compounds, food waste

## Abstract

Figs are highly perishable, with significant losses due to overripening or failure to meet market standards. Drying is essential to extending their shelf life and reducing food waste. This study evaluated the impact of traditional sun drying and hybrid solar drying on the quality of dried “Pingo de Mel” figs. Sun drying required 5–7 days, while the hybrid solar drying completed the process in 3 days. Both methods resulted in a similar final moisture content (29.43% and 28.14%, respectively), water activity (0.68 and 0.63, respectively), and hardness (2.36 and 2.61 N, respectively). Hybrid solar-dried figs exhibited slightly lower L* values and higher b* values, reflecting a darker appearance with a more pronounced yellow hue. Fresh and sun-dried figs developed fungal growth (*Alternaria* spp., *Aspergillus niger*, *Cladosporium* spp., and *Fusarium* spp.) within four weeks, while hybrid solar-dried figs remained contamination-free, improving microbial safety. Moreover, hybrid drying preserved higher levels of phenolic compounds, particularly rutin and 5-*O*-caffeoylquinic acid, along with greater antioxidant activity. Overall, hybrid solar drying offers significant advantages over traditional sun drying by reducing the drying time, enhancing microbial safety, and preserving bioactive compounds, making it a more effective method for fig preservation.

## 1. Introduction

*Ficus carica* L., commonly known as the fig, belongs to the *Moraceae* family, one of the biggest plant kingdoms, with between 600 and 2000 species [1]. The origin of the fig dates to around 9400 BCE, with numerous ancient texts, such as the Bible and Quran, describing its importance in early agricultural societies [2]. The cultivation of *F. carica* L. began in the Middle East and Southwest Asia and has since been spreading worldwide, with Turkey, Egypt, Morocco, Algeria, and Iran being the current main producers. In 2022, approximately 297,000 hectares of fig trees were harvested and over 1.2 million tons of figs were produced all over the world [3,4]. In Portugal, it represents one of the oldest fruits cultivated, and the main cultivars are “Lampa Preta”, “Pingo de Mel”, and “Figo Preto de Torres Novas”. In the 1860s, fig production played a major role in the Portuguese economy, because dried figs were used as raw material to produce alcohol and for diet consumption [3]. Yet, the production of dried figs declined, mainly due to the discovery of fully mechanized crops as new sources of raw material and the increase in labor costs [3]. Portuguese fig producers are currently very motivated to restore figs to their previous splendor, emphasizing fresh and dried figs [3]. In fact, in 2022, Portugal harvested 3620 hectares and produced 3140 tons of figs. This production is especially important due to fig trees’ resistance to climate change and the fact that they require little water to grow [3,5].

The fig fruit is an integral part of the Mediterranean diet and can be eaten fresh, dried, or in a jam. Their consumption is associated with numerous benefits, including beneficial actions in the digestive system (diabetes); gastrointestinal tract (ulcer and vomiting); respiratory system (liver disease, asthma, and cough); reproductive system (menstruation pain); infectious disease (skin disease, scabies, and gonorrhea); and even a reduction of the risk of cancer and heart disease [6,7]. In fact, figs are an excellent source of dietary fiber, minerals, vitamins, potassium, calcium, and iron, while being fat-, sodium-, and cholesterol-free and containing a high number of amino acids and antioxidants [8]. In their raw form, figs contain about 80% water, 19% carbohydrates, 1% protein, and negligible fat. After the drying process, the water content is reduced to about 30%, which increases the amount of carbohydrates to 64%, the proteins to 3%, and the fat to 1%. Dried figs are also a rich source of dietary fiber and the essential mineral manganese, while calcium, iron, magnesium, potassium, and vitamin K are present in moderate amounts [9,10,11,12].

Concerning phytochemicals, studies have reported the presence of phenolic acids, anthocyanins, proanthocyanidins, sterols, organic acids, amino acids, fatty acids, hydrocarbons, aliphatic alcohols, aldehydes, carotenoids, triterpenoids, and other secondary metabolites [13,14]. These phytochemicals are, along with fibers, the main contributors to the health benefits of fresh figs. Regarding dried figs, studies have found phytochemicals, such as flavonoids, anthocyanins, and carotenoids [13,15].

The consumption of figs is continuously increasing with consumers being interested in organically produced fruits of high quality. The increase in production has brought to light the need to extend the shelf life of these products, with drying being the most common solution. Sun drying is the traditional method for fig preservation. Although sun drying can effectively prolong the shelf life, it has several disadvantages regarding the contamination of bacteria and mold spores. In fact, the figs are not appropriately cleaned, are dried in open air, which leads to dust and dirt contamination, and are also subjected to pest infestation [16]. Additionally, fungal infections can be observed in several stages of fig growth, after the ripening of the fruit, shriveling, falling on the ground, or, as previously mentioned, during the drying process [17].

The use of hybrid solar dryers has been gaining relevance over sun drying due to allowing for (i) reduced contamination, given that there are better sanitary conditions, (ii) decreased risk of development of bacteria and fungi, (iii) accurate and controlled parameters that can be adjusted over the processing time, (iv) quickening the time of the process by increasing the air flow, (v) least dependence on weather factors, (vi) lower labor costs, and (vii) better fruit homogeneity and quality [18]. This raised the need for studies about the compositional changes that occur during the drying process and their potential impact on benefits for human nutrition and health. Additionally, it is essential to investigate how different drying methods influence the quality of the final product. This work aims to characterize the phytochemical composition of fresh figs and explore the effect of traditional sun drying versus hybrid solar drying on their phenolic profile. Furthermore, another goal of this study is to better understand the chemical and biochemical changes that happen during the drying process that may lead to changes in hardness and color. The final goal is related to the development of fungi and mycotoxins that are present in fresh figs and the influence of the drying processes in the growth of these pathogens.

## 2. Materials and Methods

### 2.1. Plant Material

*F. carica* L. (“Pingo de Mel”) samples were collected on 26 August 2024 from a cultivar located in Torres Novas, Portugal. Following collection, the samples underwent identical handling. They were transported in insulated cooling boxes to the laboratory in the Agrarian School of Polytechnic University of Viseu and stored at 5 °C overnight before being analyzed and dried on the hybrid solar dryer. The fresh fig samples used for determination of the antioxidant activity were stored at −18 °C prior to analysis, and those used for the identification and quantification of individual compounds were freeze-dried. The sun-dried figs were dried in Torres Novas under the care of local farmers.

### 2.2. Chemicals

Ethanol (analytical grade) was purchased from Fisher Scientific (Hampton, NH, USA). (±)-6-hydroxy-2,5,7,8-tetramethylchromane-2-carboxylic acid (Trolox) and 2,2-azinobis(3-ethyl-benzothiazoline-6-sulfonic acid (ABTS) were acquired from Sigma-Aldrich (St. Louis, MO, USA). Gallic acid 1-hydrate (99%), sodium carbonate anhydrous, sodium acetate anhydrous, and potassium chloride were obtained from Panreac (Barcelona, Spain). Acetonitrile of HPLC grade and acetic acid were from Fischer Scientific (Pittsburgh, PA, USA). 5′-caffeoylquinic acid was from Sigma-Aldrich (Darmstadt, Germany). Rutin and quercetin were obtained from Extrasynthese (Lyon, France).

### 2.3. Moisture

The moisture content was determined using the standard gravimetric method. Fig samples (10 g) were placed in pre-weighed containers and dried in an oven at 105 °C until a constant weight was achieved.

### 2.4. Water Activity (a_w_)

The a_w_ of the figs was measured at a controlled temperature range of 20–25 °C using a Rotronic water activity measurement device (Process Sensing Technologies, Bassersdorf, Switzerland). Data acquisition and monitoring were conducted using Rotronic HW5 software V1.0.0.

### 2.5. Firmness

The firmness of fresh figs was evaluated using the digital force gauge penetrometer PCE-FM 200 (PCE Instruments, Meschede, Germany) equipped with a 6-mm probe. Each fruit was placed on a stable surface, and the probe was pushed into the flesh until structure failure occurred. Firmness values were calculated as the average of two measurements taken on opposite sides of the fruit in the equatorial part of the fruit and expressed in newtons (N).

### 2.6. Size of the Fruits

The maximum length and width of fresh and dried figs were measured individually using a Mitutoyo digital caliper. The analysis was performed on a total of 18 samples, consisting of 6 fresh fruits, 6 sun-dried fruits, and 6 hybrid solar-dried fruits.

### 2.7. Hardness

The hardness of dried figs was analyzed using a Texture Analyser TE.XT.Plus (Stable Micro Systems, Surrey, UK), equipped with a 75-mm diameter flat compression probe (SMS P/75) (Stable Micro Systems). The operational parameters were set as follows: pre-test speed 0.50 mm/s, test speed 0.50 mm/s, post-test speed 1.00 mm/s, target mode set to a distance of 3.00 mm, a 5-s interval between cycles, trigger type set to auto (force), and a trigger force of 0.049 N [19].

### 2.8. °Brix

The °Brix value of the fresh fruit was determined using the digital refractometer ATAGO PAL-BX ACID F5 (ATAGO Co., Tokyo, Japan). A small incision was made on the fruit, and the juice was extracted and applied to the prism surface. Calibration was carried out using distilled water for auto-zero adjustment.

### 2.9. Color Analysis

Color attributes of figs were measured using a digital chromameter (PCE-CSM 5; PCE Instruments, Southampton, UK), operating with a measuring aperture of 8 mm in diameter, a geometry angle of 8°, observation angle CIE 10°, using D65 illuminant, and expressed in the CIE L*a*b* system, where L*—lightness (1–100), a*—redness (+100) and greenness (−100), and b*—yellowness (+100) and blueness (−100). The colorimeter was calibrated against a standard instrument white plate. All the samples were analyzed in triplicate.

### 2.10. Sun, Drying Process

Local farmers harvested the figs when ripened and transported them to the drying sites. In there, the fresh figs were spread on drying racks. After transferring all the fresh figs, these were covered with mesh covers to protect them from pests and dust. Through the 5 plus days that the figs were drying, they were regularly turned to ensure even drying and prevent spoilage. The dried figs were collected when the local farmers deemed them suitable to be sold.

### 2.11. Hybrid Solar Drying Process

The figs were dried in a hybrid solar dryer AVATAR 20 model (Chatron, Vale de Cambra, Portugal) installed at the School of Technology and Management of the Polytechnic University of Viseu. The dryer has a capacity of 800 L, a solar power output of 560 W, and an integrated 1 kW electrical resistance to compensate for periods of insufficient or absent solar energy. The structure is built with sandwich panels connected by zinc/steel profiles. Its vertical thermal solar panel has functional dimensions of 90 × 69 cm. Additionally, a photovoltaic panel, measuring 32 × 25.5 cm and incorporated at the center of the thermal panel, powers the inflation fan, which operates without external electrical input. The dryer is equipped with a pyrometer to monitor the outer surface temperature, as well as thermocouples and hygrometers inside to measure the internal conditions. An electrical energy accumulator meter was also included. Figs were selected according to size to ensure the same parameters, placed in trays, and dried for approximately 3 days (71 h), maintaining the temperature within the range of 45 to 55 °C [20].

### 2.12. Microbiological Evaluation

#### 2.12.1. Fungi Isolation and Replication

Figs were kept in sealed conditions at 5 °C to avoid contamination with pathogens from the outside environment. For fungi isolation, 5 figs were chosen from each of the treatments (fresh, sun-dried, and hybrid solar-dried). Isolation was carried out from small pieces of skin that were cut from sites of the fig fruit with symptoms, such as browning or cuts. The pieces of fig skin were placed directly onto potato dextrose (PDA) (42 g/L; Liofilchem Srl, Roseto degli Abruzzi, Italy) with antibiotics (streptomycin sulfate and ampicillin) and incubated at 20 ± 2 °C for 14 days. The plates were inspected daily, and any colonies grown from the fig skin fragments were replicated in PDA plates without antibiotics to establish pure colonies. This process was repeated once for fresh and hybrid solar-dried figs and thrice for sun-dried figs.

#### 2.12.2. Morphological Identification

Fungi identification was performed by examination of the mycelia and spores developed on the PDA plates under a stereomicroscope (M125; Leica Microsystems CMS, Wetzlar, Germany). The spores, conidiophores, pycnidia, and perithecia of the fungi were then examined under a microscope (Leica DM 500 with photocamera ICC50W, Heerbrugg, Switzerland).

### 2.13. Biochemical Analysis

#### 2.13.1. Extraction Procedure

Figs were subjected to a cold extraction by ethanol. This method was chosen to avoid damaging compounds. For the cold extraction, 100 mL of ethanol 70% (*v*/*v*) was added to 10 g of fig and shaken at 300 rpm for 1 h. The resulting extract was filtered twice, first with a coffee filter to remove the bigger particles and then with a G4 filter to ensure the extract was fully clean. The resulting extract was frozen at −20 °C.

#### 2.13.2. Antioxidant Capacity

In this study, two different methods were applied to measure the antioxidant capacity of the fig samples. These methods involved radical scavenging assays: 1,1-diphenyl-2-picrylhydrazyl (DPPH) and 2,2′-azinobis(3-ethylbenzothiazoline-6-sulfonic acid) (ABTS). For each of the tests, a Trolox calibration curve was established with concentrations varying from 0.005 to 0.200 mg/mL. For both methods, 50 µL of the standard/sample and 250 µL of DPPH/ABTS reagent were added into a well in the microplate. After incubation for 30 min in the dark, the absorbance was read at 517 nm (DPPH) and 734 nm (ABTS). The results were expressed in mg of Trolox equivalents (TE) per 100 g of dry fig sample (mg TE/100 g dry sample) [21].

#### 2.13.3. Analysis of Individual Compounds

Polyphenols were identified by UPLC-ESI-MS2, and their quantification was deter-mined by HPLC-DAD. Their identification was performed using an Acquity Ultra-Performance LC (UPLC) apparatus from Waters (Milford, MA, USA), and their characterization and quantification were performed using an Ultra-Fast Liquid Chromatography Prominence system (Shimadzu, Kyoto, Japan) controlled by LabSolutions software (Version 5.57; Shimadzu, Kyoto, Japan). UPLC/ESI-MS2 analysis was performed on an Acquity Ultra-Performance LC (UPLC) apparatus from Waters (Milford, MA, USA), equipped with a photodiode array detector (detection at 280, 320, 350, and 520 nm) coupled with a Bruker Daltonics (Bremen, Germany) HCT ultra-ion trap mass spectrometer with an electrospray ionization source. Separations were achieved using a (250 mm, 4 mm i.d.) Licrospher PR-18.5-mm column (Merck, Darmstadt, Germany) with a guard column operated at 30.8 °C. The mobile phase consisted of water:formic acid (99:1, mL/mL) (eluent A) and acetonitrile (eluent B). The flow rate was 1 mL/min. The elution program was as follows: 3–9% B (0–5 min); 9–16% B (5–15 min); 16–50% B (15–45 min); 50–90% B (45–48 min); 90–90% B (48–52 min); 90–3% B (52–55 min); 3–3% B (55–60 min). Samples were injected at the level of 10 μL. For polyphenol characterization, a capillary voltage of 2 kV was used in the negative ion mode. Nitrogen was used as a drying and nebulizing gas with a flow rate of 12 L/min. The de-solvation temperature was set at 365 °C and the nebulization pressure at 0.4 MPa. The ion trap was operated in Ultrascan mode from *m/z* 100 to 1000.

HPLC-DAD separations were achieved using the same column and the same gradient, and 20 μL of the samples were injected. Quantification was achieved by comparison with the standard solutions of known concentrations at 320 nm for hydroxycinnamic acid, at 350 nm for flavonols, and at 520 nm for anthocyanins. The results were expressed in mg/g of dry weight.

### 2.14. Statistical Analysis

The statistical analysis was performed by IBM SPSS Statistics Software 26.0 (IBM Corporation, New York, NY, USA) using a univariate general linear model (GLM), followed by Tukey’s HSD post hoc multiple comparisons tests, and the significance level was *p* < 0.05.

## 3. Results and Discussion

Fresh figs of the “Pingo de Mel” variety were dried using two methods: traditional sun drying and a controlled hybrid solar drying system. The hybrid system operated within a temperature range of 45 to 55 °C and significantly reduced the drying time from 120 h (five days) to approximately 71 h. This faster drying process offers a notable advantage for farmers, enabling the final product to reach the market more quickly compared to natural sun drying, thereby improving efficiency and potentially increasing profitability.

### 3.1. Quality Features of Fresh and Dried Figs

#### Physical Properties

The fresh figs had a moisture content of approximately 76% and a °Brix value of 26.87 °Brix, equivalent to about 27 g of soluble solids per 100 g of liquid (Table 1). After drying, the moisture content decreased to 28–29%, with the water activity (a_w_) of both sun-dried and hybrid solar-dried fruits measured at 0.68 ± 0.01 and 0.63 ± 0.02, respectively, consistent with their similar moisture levels. Note that a_w_, which measures the availability of water in a food product for microbial growth and chemical reactions, is a crucial parameter for ensuring food safety. In dehydrated foods, the value for the a_w_ should ideally fall between 0.60 and 0.66 to effectively inhibit the growth of bacteria, yeasts, and molds [22,23,24]. Hence, the gathered values indicate that, among the two samples, sun-dried figs are more susceptible to spoilage compared to those dried in the hybrid solar-dried system.

Hardness, in turn, represents the mechanical resistance of the fruit to deformation or penetration, making it a crucial attribute for evaluating texture and, consequently, a critical quality parameter. A good texture of both fresh and dried figs ensures a pleasant mouthfeel, transmitting the idea of the fig’s quality and ripeness. When dried figs are too hard, they might feel unappetizing, while extremely soft figs give the idea of a poorly done drying process. In this context, measuring hardness enables the standardization of product quality, ensuring it meets both consumer preferences and market expectations. Sun-dried and hybrid solar-dried figs tended to exhibit similar hardness values.

**Figure 1:**
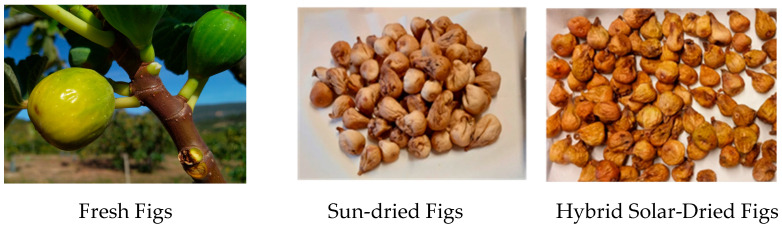
Images of fresh, sun-dried, and hybrid solar-dried figs of the “Pingo de Mel” variety. Fresh fig dimensions: 39.4 ± 5.1 mm (length) × 34.9 mm ± 3.8 (width); dried fig dimensions: 36.1 ± 3.4 mm (length) × 28.9 ± 2.1 mm (width).

Color is one of the most important quality parameters in consumer acceptance of the fruit. While fresh figs are naturally distinct in appearance from dried figs, it is notable that the two types of dried figs were also visually differentiated, with hybrid solar-dried fruits exhibiting a darker and more yellowish tone, whereas sun-dried figs appeared paler (Figure 1). This observation is consistent with the color measurements, which revealed a clear decrease in the L* (lightness) parameter for hybrid solar-dried figs compared to those dried by the sun and a higher b* value for hybrid solar-dried fruits, meaning they were yellower than the sun-dried fruits.

### 3.2. Microbiological Fungi Load

**Figure 2:**
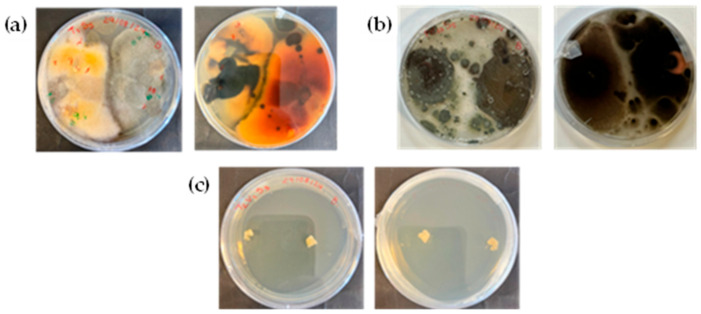
Top and bottom view of the microbial growth of fungi after 4 weeks of incubation in (**a**) fresh figs, (**b**) sun-dried figs, and (**c**) hybrid solar-dried figs.

Despite the low moisture content and a_w_ present in the two types of dried figs, fungal growth can still occur under certain conditions. These fungi may lead to the production of mycotoxins, which pose significant health risks, including acute and chronic effects such as genotoxicity and carcinogenicity, on humans. These mycotoxins are typically produced by fungi of the genera *Aspergillus*, *Penicillium*, *Fusarium*, and *Alternata* [25]. In this study, a survey was carried out to isolate and identify several fungi present in fresh, sun-dried, and hybrid solar-dried figs. After a 4-week incubation period, fungal growth was analyzed for each treatment. For fresh figs (Figure 2a), all the samples exhibited both fungal and bacterial growth. Similarly, all the sun-dried fig samples exhibited fungal growth (Figure 2b). In contrast, no pathogenic growth was observed in the hybrid solar-dried figs (Figure 2c).

**Figure 3:**
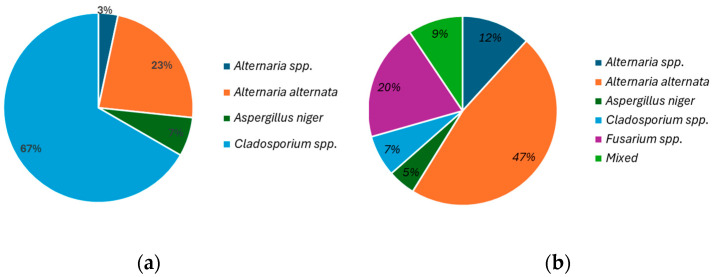
Relative percentage of each fungi identified on (**a**) fresh and (**b**) sun-dried figs.

The data revealed the presence of *Alternaria* spp., *Alternaria alternata*, *Aspergillus niger*, *Cladosporium* spp., *Fusarium* spp., and some mixed colonies. The relative percentage of each fungal genus is represented in Figure 3. A study by Galván et al. [26] also investigated mold in dried figs from distinct industrial companies. The authors reported the development of *Aspergillus* spp., *Penicillium* spp., *Cladosporium* spp., and *Alternaria* spp. Furthermore, they observed that *Aspergillus* spp. was the species that had the highest relative prevalence across most of the studied samples [26]. Among the species identified in our study, the main genera that produces mycotoxins are *Aspergillus* niger, *Alternaria* spp., and *Fusarium* spp. (Figure 3). Our study demonstrated a higher percentage of *Cladosporium* spp. in fresh figs and *Alternaria alternata* in sun-dried figs, which could be related to the fig variety analyzed or with the conditions of the environment.

**Figure 4:**
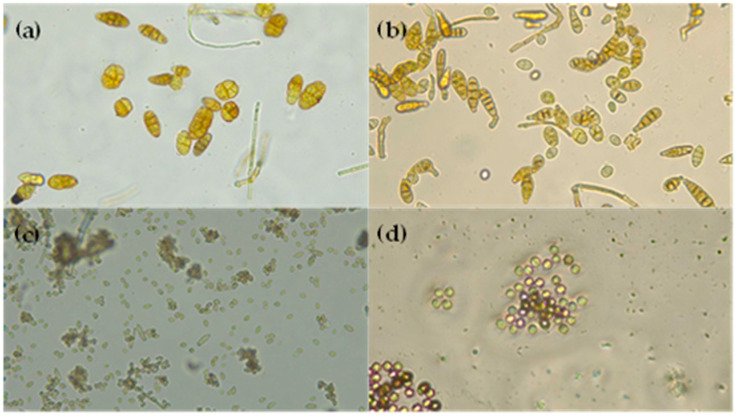
Images of (**a**) *Alternaria alternata*, (**b**) *Alternaria* spp., (**c**) *Aspergillus niger*, and (**d**) *Cladosporium* spp. taken with a microscope.

*Alternaria* species appear as green or black molds and can produce more than 70 different mycotoxins [27]. These fungi infect fruits primarily through wounds, natural openings, or compromised areas of plant tissue. Due to the soft nature of fig skin, there is a higher risk of physical damage than other kinds of fruit, which increases the risk of contamination. In this study, *Alternaria alternata* (Figure 4a) was found in 23% of fresh figs and 47% of sun-dried figs, while other *Alternaria* spp. (Figure 4b) were detected in 3% of fresh figs and 12% of sun-dried figs. Accordingly, in the study conducted by Gálvan and coworkers in three distinct industries, the authors found the presence of *Alternaria alternata* in a relative amount that ranged from 11.8% to 1.5% [26]. Further research of the same group allowed them to conclude that the relative abundance of *Alternaria alternata* generally increased after the drying process [28]. *Alternaria alternata* produces mycotoxins that are the main contaminants in agricultural products due to their high toxicological impact, including alternariol (AOH), alternariol monomethyl ether (AME), tenuazonic acid (TeA), altenuene (ALT), altertoxins (ATX), and tentoxin (TEN) [29]. López et al. observed the presence of TeA in dried figs and noted the occurrence of *Alternaria* spp. [30].

*Aspergillus* spp. (Figure 4c) appear as dark, black molds, and the mycotoxins produced by these species have the biggest impact on humans, animals, and plants health [31]. Like *Alternaria* spp., *Aspergillus* spp. infect fruits through physical damage or natural openings. Our study found the presence of *Aspergillus niger* (7% in fresh figs and 5% in sun-dried figs). This species is known to produce mycotoxins such as ochratoxin A (OTA), fumonisins, and malformins [32]. One study isolated 43 isolates of *Aspergillus niger* from dried figs [33]. Another observed that the relative abundance of *Aspergillus niger* in one case had a small increase after drying, while, in the other, was the same [28]. Furthermore, a previous study also reported the occurrence of *Aspergillus niger* in 75% of figs at the drying stage, given that this species has a tolerance against ultraviolet C due to the melanin content in their cell walls [34]. It has been reported that *Aspergillus niger* was the most common fungi among the dried fig samples [35] and that dried figs from Turkey showed a widespread infection of ochratoxin A and aflatoxin B [36]. This mycotoxin is nephrotoxic and carcinogenic, posing a significant concern for food safety.

*Cladosporium* spp. (Figure 4d) typically appear as green, olive, or brown molds. While not as hazardous as other fungi, they are considered moderately dangerous to humans, given their potential as an allergen and their association with respiratory or opportunistic infections [37,38]. In this study, *Cladosporium* spp. were found in both fresh figs (67%) and sun-dried figs (7%). Recently, Gálvan et al. analyzed dried “Calabacita” figs and found that *Cladosporium* spp. compromised 18.9% of the isolates [28]. Furthermore, they observed that certain *Cladosporium* species were present in fresh figs but were eradicated after the drying process.

*Fusarium* spp. molds are typically pink to salmon-colored, with a powdery or fluffy appearance. These species raise major concerns due to their ability to produce harmful toxins such as trichothecenes, fumonisins, and zearalenone [39,40]. In the present study, *Fusarium* spp. were detected exclusively in sun-dried figs, with a relative abundance of 20%. The presence of *Fusarium* spp. in both fresh and dried figs was previously reported, albeit at a lower relative abundance of approximately 2.5% [28].

### 3.3. Phenolic Compound Profile and Antioxidant Properties

The presence of phenolic compounds in food, namely fruit, can be important to consumers due to their health-promoting properties, which notably include antioxidant effects and others like anti-inflammatory, cardiovascular, neuroprotective, and antimicrobial benefits [33].

**Figure 5:**
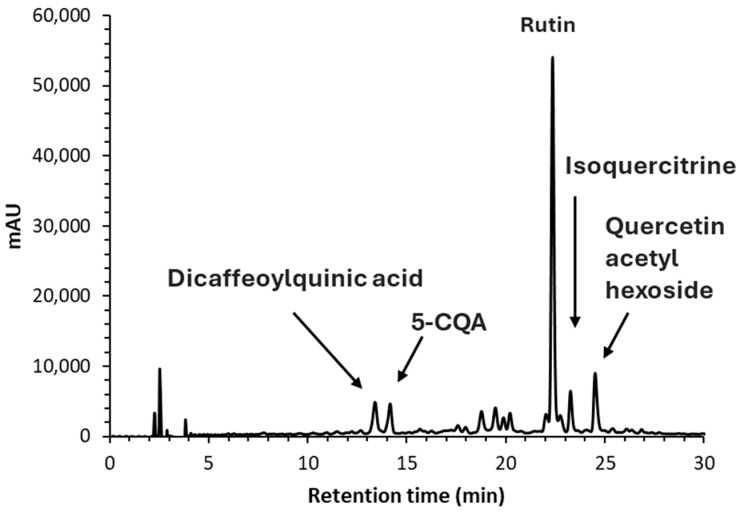
HPLC phenolic profile of fresh figs at 350 nm.

Among the detected phenolic compounds in fresh and dried figs, rutin was, by far, the most abundant, reaching 191.5 ± 12.07 mg/kg of DM in fresh figs and 48.00 ± 10.91 and 68.48 ± 1.57 mg/kg of DM in sun- and hybrid solar-dried figs, respectively (Table 2 and Figure 5). The high abundance of rutin in figs has been previously reported [15]. In addition, the identified compounds included two other quercetin derivatives and the hydroxycinnamic acids 5-*O*-caffeoylquinic acid and dicaffeoylquinic acid. Overall, the total phenolic compounds accounted for 283.74 ± 18.62 mg/kg of DM for fresh figs, decreasing by 77% in sun-dried figs (66.58 ± 13.98 mg/kg DM) and 69% in hybrid solar-dried figs (88.46 ± 2.56 mg/kg DM). The general decline in phenolic compounds may result from several factors, including (i) thermal degradation, (ii) oxidative degradation due to UV and visible light exposure, or (iii) loss of cellular integrity leading to chemical changes [41,42]. Moreover, enzymatic degradation by polyphenol oxidase (PPO) may occur. Notably, caffeoylquinic acids, particularly 5-*O*-caffeoylquinic acid, are associated with fruit browning, as they are the main substrate of PPO, which could contribute to the browning of dried “Pingo de Mel” figs [43].

Previous studies have shown that different drying techniques can significantly influence the phenolic content of dried fruits, with methods like oven and hybrid solar drying often preserving or even enhancing the phenolic levels compared to traditional sun drying. Konak et al. studied two dark-colored and two light-colored fig cultivars in both fresh and dried conditions. For the light-colored figs of the “Sarilop” and “Sarizeybek” varieties, the TPC of the sun-dried figs was 245.6 and 259.6 mg GAE/100 g DM, respectively. In turn, drying led to an increase in TPC, reaching 289.8 mg GAE/100 g DM for the “Sarilop” variety and 272.5 mg GAE/100 g DM for the “Sarizeybek” variety [44]. Similarly, Slatnar et al. studied the drying process in the “Bela petrovka” cultivar and reported findings consistent with our study, showing that the TPC levels were lower in sun-dried figs compared to oven-dried figs [18].

Overall, the antioxidant capacities of the figs followed the same trend as their phenolic compound content (Table 2 versus Table 3): fresh > hybrid solar-dried > sun-dried. Specifically, the scavenging abilities of ABTS and DPPH radicals in fresh figs were 137.7 mg TE/100 g DM and 50.0 mg TE/100 g DM, respectively. The hybrid solar-dried figs showed a decrease of 29% for ABTS (98.2 mg TE/100 g DM) and 49% for DPPH (25.5 mg TE/100 g DM), while sun-dried figs exhibited even lower values, with decreases of 68% for ABTS (44.1 mg TE/100 g DM) and 81% for the DPPH assay (9.6 mg TE/100 g DM).

Konak et al. determined the antioxidant activity of the light-colored “Sarilop” and “Sarizeybek” figs varieties by the ABTS radical cation decolorization assay. Their findings indicated that the sun-dried “Sarilop” figs exhibited higher antioxidant activity, whereas oven-dried “Sarizeybek” figs had a higher antioxidant activity [44]. Slatnar et al. studied the antioxidant activity through the DPPH radical scavenging method and found that both drying methods provided similar antioxidant activities [18].

## 4. Conclusions

This paper focused on the comparison of two drying methods for “Pingo de Mel” figs, highlighting the differences in quality attributes, chemical composition, texture properties, fungal growth, phenolic content, and antioxidant activity. Traditional sun drying tends to have a higher microbial load and longer drying times. On the other hand, hybrid solar drying offers faster processing, better microbial safety, consistent moisture content, increased preservation of the phenol content, and higher antioxidant activity. This study highlights the benefits of a hybrid solar drying system, offering a reduced drying time, enhanced food safety, and better retention of quality attributes. These advantages make it a highly efficient alternative to traditional sun drying for extending the shelf life of figs. While the hybrid solar dryer system offers many strengths, certain limitations should be considered. These include the initial investment required for installation, ongoing maintenance and operational costs, and the need for technical skills to ensure optimal performance. To further advance the application of hybrid solar drying, future research could evaluate its impact on sugar concentrations, prebiotic compounds, and vitamin retention. Additionally, studies assessing the long-term effects on shelf life, consumer acceptance, and economic feasibility would provide valuable insights for more widespread adoption.

## Figures and Tables

**Table 1:** Physical features of fresh and dried “Pingo de Mel” figs.

		Fresh	Sun-Dried	Hybrid Solar-Dried
Moisture (%)		75.66 ± 0.20 ^a^	29.43 ± 0.06 ^b^	28.14 ± 0.08 ^b^
Brix		26.87 ± 3.63	ND	ND
a_w_		ND	0.68 ± 0.01 ^a^	0.63 ± 0.02 ^b^
Firmness (N)		2.83 ± 0.85	ND	ND
Hardness (N)		ND	2.36 ± 0.48 ^a^	2.61 ± 0.51 ^a^
	a*	−8.07 ± 3.59 ^a^	11.54 ± 1.03 ^b^	10.34 ± 3.11 ^b^
Color	b*	56.72 ± 4.22 ^a^	31.39 ± 2.77 ^b^	38.06 ± 7.86 ^b^
	L*	47.77 ± 8.77 ^a^	60.89 ± 3.48 ^b^	69.08 ± 3.29 ^c^

ND—not determined. Values are the means of the replicates and standard deviations. Values in the same column with different superscripts (a, b, and c) are significantly different at *p* < 0.05.

**Table 2:** Contents of the main phenolic compounds of fresh and dried “Pingo de Mel” figs (in mg/kg DM).

[M-H]^−^	MS2	UV Max	Compound	Fresh	Sun-Dried	Hybrid Solar-Dried
515	353, 191	317	Dicaffeoylquinic acid	5.04 ± 0.11 ^a^	1.81 ± 0.13 ^b^	0.80 ± 0.22 ^c^
353	191	325	5-*O*-caffeoylquinic acid	32.49 ± 2.09 ^a^	5.30 ± 0.36 ^b^	11.33 ± 0.23 ^c^
609	301	256, 354	Rutin	191.45 ± 12.07 ^a^	48.00 ± 10.91 ^b^	68.48 ± 1.57 ^c^
463	301	256, 353	Isoquercitrin	17.89 ± 1.71 ^a^	4.09 ± 0.91 ^b^	3.43 ± 0.43 ^b^
505	301, 463	256, 353	Quercetin acetyl hexoside	36.87 ± 2.63 ^a^	7.38 ± 1.68 ^b^	4.41 ± 0.10 ^c^
Total Phenolic Compounds				283.74 ± 18.62 ^a^	66.58 ± 13.98 ^b^	88.46 ± 2.56 ^c^

Values are the means of the replicates ± standard deviations. Values in the same column with different superscripts (a, b, and c) are significantly different at *p* < 0.05.

**Table 3:** Antioxidant activity of fresh and dried “Pingo de Mel” figs.

	Fresh	Sun-Dried	Hybrid Solar-Dried
ABTS (mg TE/100 g DM)	137.7 ± 10.8 ^a^	44.1 ± 2.0 ^b^	98.2 ± 6.7 ^c^
DPPH (mg TE/100 g DM)	50.0 ± 12.1 ^a^	9.6 ± 2.7 ^b^	25.5 ± 4.0 ^c^

Results are expressed in mg Trolox equivalents per 100 g of dry mass (mg TE/100 g DM) and are presented as mean values ± standard deviations. The analyses consisted of three replicates and were performed in triplicate. Values in the same column with different superscripts (a, b, and c) are significantly different at *p* < 0.05.

## Data Availability

The raw data supporting the conclusions of this article will be made available by the authors on request.
